# Mapping Oat Crown Rust Resistance Gene *Pc45* Confirms Association with *PcKM*

**DOI:** 10.1534/g3.118.200757

**Published:** 2018-12-15

**Authors:** Aida Z. Kebede, Jayelle Friesen-Enns, Belaghihalli N. Gnanesh, Jim G. Menzies, Jennifer W. Mitchell Fetch, James Chong, Aaron D. Beattie, Edyta Paczos-Grzęda, Curt A. McCartney

**Affiliations:** * Morden Research and Development Center, Agriculture and Agri-Food Canada, Morden, MB, Canada; † Brandon Research and Development Center, Agriculture and Agri-Food Canada, Brandon, MB, Canada; ‡ Crop Development Centre/Department of Plant Sciences, University of Saskatchewan, Saskatoon, SK, Canada; § Institute of Plant Genetics, Breeding and Biotechnology, University of Life Sciences, Lublin, Poland

**Keywords:** *Pc45*, oat, *Avena sativa*, crown rust, Puccinia coronata, *PcKM*, linkage

## Abstract

Molecular mapping of crown rust resistance genes is important to effectively utilize these genes and improve breeding efficiency through marker-assisted selection. *Pc45* is a major race-specific crown rust resistance gene initially identified in the wild hexaploid oat *Avena sterilis* in the early 1970s. This gene was transferred to cultivated oat (*Avena sativa*) and has been used as a differential for identification of crown rust races since 1974. Previous research identified an association between virulence to *Pc45* and *PcKM*, a crown rust resistance gene in the varieties ‘Kame’ and ‘Morton’. This study was undertaken to reveal the relationship between *Pc45* and *PcKM*. *Pc45* was studied in the crosses ‘AC Morgan’/*Pc45* and ‘Kasztan’/*Pc45*, where *Pc45* is the differential line carrying *Pc45*. F_2_ progenies and F_2:3_ families of both populations were inoculated with the crown rust isolate CR258 (race NTGG) and single gene segregation ratios were observed. SNP markers for *PcKM* were tested on these populations and linkage maps were generated. In addition, 17 newly developed SNP markers identified from genotyping-by-sequencing (GBS) data were mapped in these two populations, plus another three populations segregating for *Pc45* or *PcKM*. *Pc45* and *PcKM* mapped to the same location of Mrg08 (chromosome 12D) of the oat chromosome-anchored consensus map. These results strongly suggest that *Pc45* and *PcKM* are the same resistance gene, but allelism (*i.e.*, functionally different alleles of the same gene) or tight linkage (*i.e.*, two tightly linked genes) cannot be ruled out based on the present data.

Oat (*Avena sativa* L.) is an economically important cereal crop highly valued for its health benefits. The estimated global oat production was 23.6 million tons on 9.6 million hectares in 2017 (USDA/Foreign Agricultural Service 2018; https://www.fas.usda.gov/data/world-agricultural-production). One third of this production was in North America, with Iowa, Minnesota, Wisconsin, North and South Dakota, and the three Canadian prairie provinces of Alberta, Manitoba and Saskatchewan, being significant oat growing regions (Menon *et al.* 2016). In these regions, productivity over the years has been challenged by the frequent occurrence of crown rust caused by *Puccinia coronata* Corda f. sp. *avenae* Eriks. (Carson 2011, Chong *et al.* 2011). Crown rust affects the photosynthetic ability of the plant by damaging the leaf tissue, thereby reducing grain filling and lowering grain yield.

The most effective, economical, and environmentally friendly method of controlling crown rust disease is the deployment of resistant cultivars. Success in resistant cultivar development depends on thorough knowledge of the genetics of host resistance. Crown rust resistance can be qualitative (*i.e.*,major gene; resistance based on a gene-for-gene interaction) or quantitative (*i.e.*, minor or major gene; non-race-specific partial resistance). Although both resistance mechanisms are effective in controlling the disease, most breeding programs commonly use major gene resistance. Major gene resistance is simpler to utilize in breeding programs compared to minor gene resistance because it is easier to select in greenhouse seedling tests or in field nurseries.

The effectiveness of a newly deployed major gene is usually limited to two to five years (Carson 2011). New races evolve through a combination of sexual recombination on the alternate host and mutation to overcome individually deployed major resistance genes. The presence of the alternate host common buckthorn (*Rhamnus cathartica*) in the vicinity of oat fields accelerates the evolution of new virulent races (Zhao *et al.* 2016). Pyramiding multiple major resistance genes into a single cultivar may increase the effective life span of resistant genes. However, introgression of several effective major genes into a single cultivar is challenging when rust isolates with the necessary virulence/avirulence combinations to detect the presence of all the genes in the pyramid are unavailable. Molecular markers offer the most convenient tool for pyramiding multiple resistance genes into a single cultivar.

There are close to 100 *Pc* (*Puccinia coronata* reaction) genes identified for crown rust resistance (Simons *et al.* 1978; Crown rust resistance genes catalog: https://www.ars.usda.gov/midwest-area/stpaul/cereal-disease-lab/docs/resistance-genes/resistance-genes/). Linkage or allelic relationship has been reported among *Pc* genes from cultivated oat or wild oat relatives but allelic relationships are not well understood (Gnanesh *et al.* 2014). Further research is needed to clarify allelic relationships among the known *Pc* genes. Several types of molecular markers have been used to map *Pc* genes. RFLP and RAPD markers were used to map numerous *Pc* genes including *Pc38*, *Pc39*, *Pc48*, *Pc71*, *Pc91* and *Pc92* (Bush and Wise 1998, Penner *et al.* 1993, Rooney *et al.* 1994, Wight *et al.* 2004, Wilson and McMullen 1996). DArT markers were used to map *Pc91* (McCartney *et al.* 2011), which were later converted into KASP markers for use in marker assisted breeding (Gnanesh *et al.* 2013).With the current availability of the oat consensus genetic map (Chaffin *et al.* 2016) and high throughput genotyping systems, such as the 6K Infinium iSelect (Tinker *et al.* 2014) and GBS SNPs (Huang *et al.* 2014), the genetic locations of *Pc38*, *Pc48*, *Pc54*, *Pc58a*, *Pc59*, *Pc68*, *Pc71*, *Pc91*, and *PcKM* were confirmed (Esvelt Klos *et al.* 2017). Nevertheless, there are still several *Pc* genes not yet mapped that could be allelic with previously mapped genes.

To date, 96 *Pc* genes have been designated with a permanent gene number. *PcKM* is a temporary designation of a major crown rust resistance gene that was first characterized in the oat cultivars Kame and Morton (Gnanesh *et al.* 2015, McMullen *et al.* 2005). It was mapped to Mrg08 (Chromosome 12D) of the oat chromosome-anchored linkage map and KASP markers closely linked to this gene were developed. Gnanesh *et al.* (2015) proposed that *PcKM* was *Pc45* based upon haplotype analysis of *PcKM*-linked SNPs and comparison of the individual *P. coronata* races to which *PcKM* and *Pc45* confer resistance. To further investigate whether *PcKM* and *Pc45* are the same gene, crown rust resistance in a *Pc45* differential line was mapped in crosses involving two susceptible cultivars, AC Morgan (Kibite and Menzies 2001) and Kasztan (Paczos-Grzęda 2004).

The current paper reports genetic mapping of the major crown rust resistance gene *Pc45* in two bi-parental populations using seedling reactions to rust isolate CR258 (race NTGG) and linkage maps constructed with KASP marker data. The genetic locations of *Pc45* and *PcKM* were compared to determine if the two genes are allelic or tightly linked. In addition, predictive GBS-derived KASP markers were validated on a set of diverse oat lines.

## Materials and Methods

### Germplasm

Crosses were made between the *Pc45* differential line (Pendek*4/CAV 5050) with two crown rust susceptible cultivars, AC Morgan and Kasztan. The AC Morgan/*Pc45* population consisted of 246 F_2:3_ families, while the Kasztan/*Pc45* population consisted of 76 F_2_ individuals of which 73 produced enough seed to be phenotyped as F_2:3_ families. The *Pc45* differential line was developed from a cross between the *A. sterilis* line, F-169 (CAV 5050), collected from Israel (Fleischmann *et al.* 1971) to a universally susceptible line Pendek. Three backcrosses were made to the susceptible parent to ensure the line carried only the resistant gene of interest. AC Morgan is a high yielding Canadian cultivar developed from the cross OT526/OT763 (Kibite and Menzies 2001) that lacks any major *Pc* genes. Kasztan is a Polish cultivar developed from the cross Dawid/CHD 1685/84 (Paczos-Grzęda 2004). The susceptible cultivars provided two genetically diverse backgrounds to map *Pc45*. Three RIL populations (OT3019/Morton, 100 RILs; CDC Weaver/Kame, 72 RILs; OT9001/OT3060, 72 RILs) previously used to map *PcKM* (Gnanesh *et al.* 2015) were also used in this study. A subset of 71 oat lines from the Collaborative Oat Research Enterprise (CORE) project (Esvelt Klos *et al.* 2017) was used as a haplotype panel to identify predictive SNPs for use in oat breeding.

### Crown rust inoculation and disease rating

Seedlings of parental lines, F_2_ progeny (from a single F_1_ plant for each cross) and F_2:3_ families from the AC Morgan/*Pc45* and Kasztan/*Pc45* crosses and the haplotype panel of 71 lines were inoculated at the one leaf stage with *P. coronata* isolate CR258 (race NTGG), which has the virulence/avirulence formula of *Pc38*, *39*, *40*, *46*, *48*, *52*, *56*, *68/45*, *50*, *51*, *54*, *58*, *59*, *62*, *64*, *91*, *94*, *96* (Green 1965). The rust inoculum was prepared by first heat shocking the vial containing frozen urediniospores (-80° freezer) in a water bath at 40° for five min. In each inoculum capsule, 4 mg spores were suspended in 450 ml light mineral oil (Bayol, Esso Canada, Toronto, ON). The urediniospores were uniformly sprayed over the seedlings using a custom made micro inoculator. After inoculation, the oil was allowed to evaporate for 30 min and seedlings were placed in dew chambers (Model I-60D, Percival Scientific, Perry, Iowa) overnight in the dark with a constant temperature of 18°. The inoculated seedlings were then transferred to a greenhouse with a 18 - 21° temperature and 16 hr of light supplemented by fluorescent lighting (Canopy level light intensity = 450 μmol m^-2^ s^-1^). Infection type (IT) data were scored 10-12 days after inoculation using the 0 – 4 scale (Murphy 1935) where 0 (immune), ; (fleck), 1 and 2 were considered as resistant (R), while 3 and 4 were susceptible (S). For F_2_ progeny, IT data were recorded on single plants. For F_2:3_ families, IT data of 20 – 24 plants from each family were recorded to differentiate homozygous resistant, segregating and homozygous susceptible families. For the haplotype panel of 71 oat lines, IT data were recorded based on the reaction of four plants per line. To assess the reaction of *Pc45* and *PcKM* to diverse crown rust race isolates, seedlings of oat lines AC Morgan, Morton and *Pc45* were evaluated with CR258 and seven additional isolates (CR13, CR223, CR241, CR249, CR254, CR257, CR259) using the same inoculation protocol and 0 – 4 disease scoring scale as the *Pc45* mapping population. The virulence formulae of all *P. coronata* isolates are detailed in Table S2.

### DNA extraction and marker development

Tissue samples for DNA extraction were collected from F_2_ plants at the 3 – 4 leaf stage after scoring infection types and removing infected leaves. The youngest leaf (the uppermost 3^rd^ or 4^th^ leaf) tissues were collected in tubes, lyophilized (approximately 10 grams dry weight) and stored at -80° until used. High quality DNA was extracted from the freeze dried leaf tissues using the DNeasy Plant DNA extraction kit (Qiagen, Toronto, Canada). DNA was quantified using the blue fluorescent dye Hoechst 33258 and read using a Tecan Infinite F500 (Tecan Group Ltd. Männedorf, Switzerland).

Kompetitive Allele Specific PCR (KASP) markers were used to map *Pc45* (Table S1). Each KASP marker had two allele specific forward primers and two common reverse primers designed. Each forward primer had a distinct fluorescent dye associated with it. The KASP assay consisted of 2.5 μl of DNA (15 ng/μl), 2.5 μl of 2× KASP reaction mix and 0.07 μl of primer mix. Each primer mix contained two forward primers and one reverse primer at 100pmol/μl concentration. The second reverse primer was only used if the first reverse primer did not work. Polymerase Chain Reaction (PCR) was performed in a 5μl reaction volume. PCR conditions consisted of an initial denaturation at 94° for 15 min, 94° for 20 sec, 10 touchdown cycles over 61-55° for 1 min (dropping down by 0.6° per cycle) and 26 cycles of 20 s at 94° followed by an extension for 1 min at 55°. Following the PCR reaction, samples were incubated at 4° until fluorescence was quantified with an Omega Fluorostar scanner (BMG LABTECH GmbH, Offenburg, Germany). The fluorescence data were analyzed using KlusterCaller software (LGC Genomics LLC, Beverly, MA, USA).

The parental lines used to map *Pc45* and a subset of 14 RILs from the Morton/OT3019 population (used to map *PcKM*) were genotyped using genotyping-by-sequencing (GBS) following the protocol described by Huang *et al.* (2014). Briefly, DNA was digested using two restriction enzymes *Pst*I (CTGCAG) and *Msp*I (CCGG), barcoded adaptors were then ligated to each DNA sample from a 96 well plate, pooled into a single library and PCR amplified. The pooled library was sequenced on a single lane of HiSeq 2500 (Illumina, San Diego, CA, USA). The single-end sequenced reads were 150 bases in length. SNP calling from the sequenced reads was performed using Haplotag software (Tinker *et al.* 2016). A total of 240 K haplotype-based SNPs were called.

The GBS SNPs for *Pc45*/*PcKM* linkage map construction were 14 SNPs selected based on occurrence on the linkage group Mrg08 and polymorphism between parents of *Pc45*/*PcKM* linkage mapping populations. The linkage group, Mrg08 used as reference to select these SNPs was constructed from linkage map constructed using AC Morgan / CDC Morrison F_6:8_ RILs (Unpublished data). Therefore, the Mrg08 linkage group used in the current study has additional SNPs that are not yet mapped in the consensus genetic map of Chaffin *et al.* (2016).

The 14 Morton/OT3019 RILs (7 resistant and 7 susceptilble RILs) were used to identify polymorphic SNPs linked to *PcKM*. These SNPs were subsequently used for linkage map construction. We also used an additional 17 KASP markers developed in a previous study for mapping the *PcKM* gene in cultivars Kame and Morton (Gnanesh *et al.* 2015). The *PcKM* linked KASP markers were designed from GBS SNPs (Huang *et al.* 2014), 6K oat Infinium SNPs (Tinker *et al.* 2014) and a RAPD fragment (Gnanesh *et al.* 2015).

### Statistical analyses

Crown rust IT data were tested for goodness-of-fit with expected single gene segregation ratios for F_2_ progeny (3 resistant: 1 susceptible) and F_3_ families (1 homozygous resistant: 2 segregating: 1 homozygous susceptible) using χ^2^ tests.

MapDisto software (Lorieux 2012) was used to construct linkage maps. Genotypes and markers with >10% missing marker data were filtered out of the analysis. Linkage groups were determined based on a minimum LOD (logarithm of odds) score of 3 and a maximum recombination fraction of 0.3. Map distances were calculated using the Kosambi mapping function (Kosambi 1944).

### Data availability

Table S1 outlines the KASP markers and their primer sequences used to map the *Pc45* gene. Table S2 describes the virulence formulae of *P. coronata* isolates used in this study. Supplemental material is available at Figshare: https://figshare.com/s/e43146cbee335e0aa147. Supplemental material available at Figshare: https://doi.org/10.25387/g3.7393400.

## Results

AC Morgan and Kasztan had susceptible infection types (IT scores = 3 – 4) while the *Pc45* differential line had a highly resistant reaction (IT score = 0) toward crown rust race NTGG. There were 246 individual F_2_ plants tested from the AC Morgan/*Pc45* cross and 76 from the Kasztan/*Pc45* cross (Table 1). The population size was lower for the Kasztan/*Pc45* cross because of poor germination of the F_2_ seeds. The segregation ratios obtained from the F_2_ progeny and F_2:3_ families for all crosses were not significantly different from that expected for a single gene (Table 1). This indicated that a single dominant gene controlled resistance to race NTGG in both populations.

**Table 1:** Segregation ratios of F_2_ progeny and F_2:3_ families from the crosses AC Morgan/*Pc45* and Kasztan/*Pc45* when inoculated with crown rust isolate CR258^#^ (race NTGG*)

Population	Generation	Resistant	Segregating	Susceptible	Ratio	χ^2^	*P-Value*
AC Morgan/*Pc45*	F_2_	178	—	68	3:1	0.92	0.34 ns
	F_2:3_	58	120	68	1:2:1	0.96	0.62 ns
Kasztan/*Pc45*	F_2_	57	—	19	3:1	0.00	1.00 ns
	F_2:3_	18	36	19	1:2:1	0.04	0.98 ns

ns = non-significant; ^#^Designated crown rust isolate numbers at Morden Research and Development Centre; *Race designation according to the standard North American system of nomenclature for *Puccinia coronata* (Chong *et al.* 2000) using 16 primary crown rust differentials (set 1: *Pc40*, *45*, *46*, *50*; set 2: *Pc38*, *39*, *48*, *68*; set 3: *Pc51*, *52*, *58*, *59*; set 4: *Pc54*, *56*, *62*, *64*) and three supplemental differentials (*Pc91*, *94*, *96*).

The *Pc45* differential line and Morton (source of *PcKM*, McMullen *et al.* 2005) were inoculated with an additional seven crown rust isolates (Table 2). Both lines had a similarly low infection type to six of the seven isolates. A high infection type was observed for isolate CR13 (race SJGL-96) for both Morton and the *Pc45* differential line. Isolate CR13 has the virulence/avirulence pattern of *Pc39*, *40*, *45*, *46*, *48*, *52*, *54*, *96*/*38*, *50*, *51*, *56*, *58*, *59*, *62*, *64*, *68*.

**Table 2:** Infection type for AC Morgan, the *Pc45* differential line and Morton to a set of diverse crown rust isolates

Genotype	Crown Rust Isolate^#^ (Race*)							
	13 (SJGL-96)	223 (NGCB-94)	241 (DSGB)	249 (DQBG)	254 (BRBG)	257 (BRBG-94)	258 (NTGG)	259 (LQCB-91)
AC Morgan	4	4	4	4	4	4	4	4
*Pc45*	4	;	;	0	0	;	0	;(;1)
Morton	4	;-	;-	0	0;-	0;-	0	0;-

^#^ Designated crown rust isolate numbers at Morden Research and Development Centre;

* Race designation according to the standard North American system of nomenclature for *Puccinia coronata* (Chong *et al.* 2000) using 16 primary crown rust differentials (set 1: *Pc40*, *45*, *46*, *50*; set 2: *Pc38*, *39*, *48*, *68*; set 3: *Pc51*, *52*, *58*, *59*; set 4: *Pc54*, *56*, *62*, *64*) and three supplemental differentials (*Pc91*, *94*, *96*).

**Figure 1:**
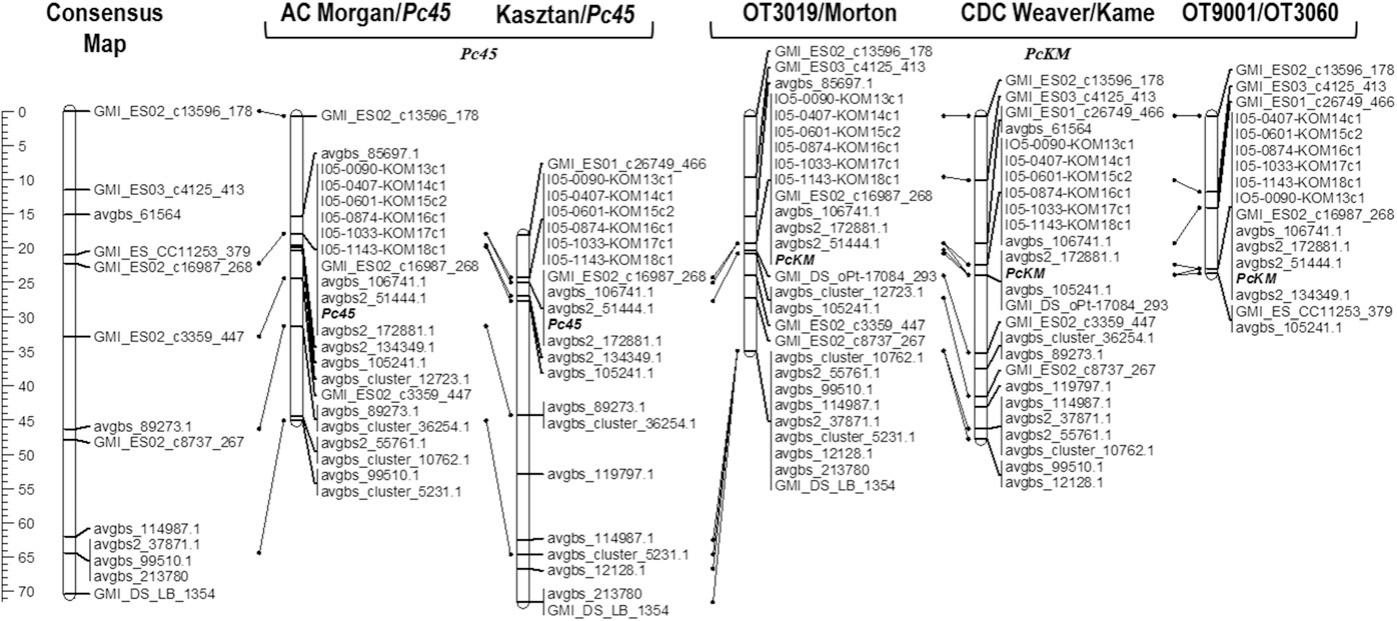
Linkage maps showing the common location of the *Pc45* and *PcKM* crown rust resistance genes. The “Consensus Map” refers to Mrg08 (Chromosome 12D) of the chromosome anchored consensus genetic map (Chaffin *et al.* 2016).

Fourteen polymorphic SNPs identified from the GBS data were used for KASP assay design. Four of these 14 SNPs were previously mapped in the consensus framework map and published by Chaffin *et al.* (2016), while the remaining ten were novel. Among the 17 KASP markers previously used to map *PcKM*, five were polymorphic in the AC Morgan/*Pc45* cross and six were polymorphic in the Kasztan/*Pc45* cross (Figure 1). Four additional markers, I05-0407-KOM14, I05-0601-KOM15, I05-1033-KOM17 and I05-1143-KOM18 were designed from the same RAPD fragment used to develop I05-0090-KOM13 and I05-0874-KOM16 (Gnanesh *et al.* 2015) and mapped in the AC Morgan/*Pc45* and Kasztan/*Pc45* cross. The three *PcKM* linkage maps were updated with the additional I05-derived markers and the newly designed GBS-derived KASP markers, which improved map density and allowed comparison of the map locations of *Pc45* and *PcKM* across the five populations. The order of loci was consistent in the AC Morgan/*Pc45* and Kasztan/*Pc45* populations and with the three populations used for *PcKM* mapping. All five maps were also consistent with the oat consensus genetic maps developed by Oliver *et al.* (2013) and Chaffin *et al.* (2016).

The genetic linkage maps placed the *Pc45* gene between 80.2 and 81.4 cM on Mrg08 (chromosome 12D) of the chromosome-anchored oat consensus linkage map (Figure 1). The genetic location of *Pc45* could not be differentiated from the genetic location of *PcKM*. The best diagnostic marker identified for *PcKM* in an earlier study by Gnanesh *et al.* (2015), I05-0874-KOM16c1 was also one of the closest linked markers to *Pc45*. There were no GBS markers identified from the AC Morgan/CDC Morrison RIL population that co-localized with *Pc45*, but three new GBS markers (avgbs2_51444.1, avgbs2_172881.1 and avgbs_106741.1) were identified using the 14 RIL subset of the Morton/OT3019 population. These three GBS-derived SNP markers were genotyped as KASP assays and co-localized with *Pc45/PcKM* in the five mapping populations.

A total of 71 diverse oat lines from the CORE project were tested with the KASP markers developed by Gnanesh *et al.* (2015) (I05-0090-KOM13c1, I05-0874-KOM16c1, GMI_ES02_c16987_268, GMI_DS_oPt-17084_293) and with the newly discovered GBS SNPs (avgbs2_51444.1, avgbs2_172881.1 and avgbs_106741.1). The co-dominant KASP markers I05-0090-KOM13c1 and I05-0874-KOM16c1 were the most predictive assays for *Pc45* and *PcKM* (Table 3). Avgbs2_172881.1 was the only marker that co-segregated with *Pc45* and *PcKM* in all the crosses tested, although it is a dominant marker.

**Table 3:** Evaluation of the predictive ability for markers linked to the *Pc45/PcKM* gene using a diverse set of oat lines

Oat line	IT*	*Pc45/PcK*M status	I05-0090-KOM13c1	I05-0874-KOM16c1	GMI_ES02_ c16987_268	GMI_DS_oPt-17084_293	avgbs2_ 51444.1	avgbs2_ 172881.1	avgbs_ 106741.1
*Pc45*	0	Carrier	A	A	A	A	A	A	A
Morton	0	Carrier	A	A	A	A	A	A	A
Weaver	4	Non-carrier	B	B	B	B	B	B	B
AC Morgan	4	Non-carrier	B	B	B	A	B	B	B
Kasztan	4	Non-carrier	B	B	B	A	B	B	B
00Ab7006	4	Non-carrier	B	B	B	A	B	B	B
00Ab7085	4	Non-carrier	B	B	B	A	B	B	B
02Ab6655	4	Non-carrier	B	B	B	A	B	B	B
02HO-139	4	Non-carrier	B	B	B	A	B	B	B
95Ab12770	4	Non-carrier	B	B	B	A	B	B	B
95Ab13050	4	Non-carrier	B	B	B	A	B	B	B
98Ab7265	4	Non-carrier	B	B	B	A	B	B	B
99Ab10937	4	Non-carrier	B	B	B	A	B	B	B
99Ab10987	4	Non-carrier	B	B	B	A	B	B	B
99Ab11098	4	Non-carrier	B	B	B	A	B	B	B
99Ab11227	4	Non-carrier	B	B	B	A	B	B	B
99Ab11391	4	Non-carrier	B	B	B	A	B	B	B
Andrew	4	Non-carrier	B	B	B	A	B	B	B
ARDENTE	4	Non-carrier	B	B	B	A	B	B	B
BARRA	4	Non-carrier	B	B	B	A	B	B	B
Biri	4	Non-carrier	B	B	B	A	B	B	B
Bond	4	Non-carrier	B	B	B	A	B	B	B
Brown 1409-164	4	Non-carrier	B	B	B	A	B	B	B
Bullion	4	Non-carrier	B	B	B	A	B	B	B
CILLA	4	Non-carrier	B	B	B	A	B	B	B
CIRCLE	4	Non-carrier	B	B	B	A	B	B	B
Clintland 60	4	Non-carrier	B	B	B	A	B	B	B
Clinton 59	4	Non-carrier	B	B	B	B	B	B	B
Columbia	4	Non-carrier	B	B	B	A	B	B	B
Derby	4	Non-carrier	B	B	B	A	B	B	B
Firth	4	Non-carrier	B	B	B	A	B	B	B
FREJA	4	Non-carrier	B	B	B	A	B	B	B
Gere	4	Non-carrier	B	B	B	A	B	B	B
GN04399	4	Non-carrier	B	B	B	A	B	B	B
Grane	4	Non-carrier	B	B	B	A	B	B	B
Grenader	4	Non-carrier	B	B	B	A	B	B	B
GUNHILD	4	Non-carrier	B	B	B	A	B	B	B
HA05AB34-48	4	Non-carrier	B	B	B	A	B	B	B
HA05AB35-16	4	Non-carrier	B	B	B	A	B	B	B
HA05AB38-22	4	Non-carrier	B	B	B	A	B	B	B
HA05AB41-38	4	Non-carrier	B	B	B	A	B	B	B
HA05AB42-20	4	Non-carrier	B	B	B	A	B	B	B
HA05AB53-40	4	Non-carrier	B	B	B	A	B	B	B
HA05AB9-52	4	Non-carrier	B	B	B	A	B	B	B
HA08-03X31-1	4	Non-carrier	B	B	B	A	B	B	B
IL02-5630	4	Non-carrier	B	B	B	A	B	B	B
IL2250-14 (PI641977)	4	Non-carrier	B	B	B	A	B	B	B
Kapp	4	Non-carrier	B	B	B	A	B	B	B
Kolbu	4	Non-carrier	B	B	B	A	B	B	B
LAO-1134-022	4	Non-carrier	B	B	B	A	B	B	B
LAO-1136-024	4	Non-carrier	B	B	B	A	B	B	B
LAO-1136-056	4	Non-carrier	B	B	B	A	B	B	B
Lena	4	Non-carrier	B	B	B	A	B	B	B
Lennon	4	Non-carrier	B	B	B	A	B	B	B
LIPOPLUS	4	Non-carrier	B	B	B	A	B	B	B
Moholt	4	Non-carrier	B	B	B	A	B	B	B
ND001397	4	Non-carrier	B	B	B	A	B	B	B
Nes	4	Non-carrier	B	B	B	A	B	B	B
Nudist	4	Non-carrier	B	B	B	A	B	B	B
Odal	4	Non-carrier	B	B	B	A	B	B	B
Ogle	4	Non-carrier	B	B	B	A	B	B	B
Olram	4	Non-carrier	B	B	A	A	A	A	A
PI266887-1	4	Non-carrier	B	B	B	A	B	B	B
Pl263412-1	4	Non-carrier	B	B	B	A	B	B	B
POL	4	Non-carrier	B	B	B	A	B	B	B
Porter	4	Non-carrier	B	B	B	A	B	B	B
Racoon	4	Non-carrier	B	B	B	A	B	B	B
Ringsaker	4	Non-carrier	B	B	B	A	B	B	B
SA070592	4	Non-carrier	B	B	B	B	B	B	B
SW KERSTIN	4	Non-carrier	B	B	B	A	B	B	B
SW VAASA	4	Non-carrier	B	B	B	A	B	B	B
Tippecanoe	4	Non-carrier	B	B	B	A	B	B	B
Tyler	4	Non-carrier	B	B	B	A	B	B	B
VAO-58	4	Non-carrier	B	B	B	A	B	B	B
X8826-1	4	Non-carrier	B	B	B	A	B	B	B
Zuton	4	Non-carrier	B	B	B	A	B	B	B

* IT, infection type scores were based on inoculation with oat crown rust isolate CR258 (race NTGG).

## Discussion

This study mapped the *Pc45* gene on the Mrg08 linkage group of the oat consensus genetic map at the same location as *PcKM*. The genetic location was consistent in both the AC Morgan/*Pc45* and Kasztan/*Pc45* populations. This result strongly suggests that *Pc45* and *PcKM* are the same gene. Although both genes mapped to the same location this is not concrete evidence for allelism as tight linkage is still a possibility.

The majority of crown rust resistance genes currently identified including *Pc45* and *PcKM* originate from *A. sterilis* because its ploidy level is the same as cultivated oat, *A. sativa*, which makes it relatively easy to transfer resistance genes with conventional breeding methods (Martens and Dyck 1989). There have been few studies conducted to determine allelic relationships among the 90 published (out of the 96 designated) *Pc* genes. The traditional way of differentiating linkage from allelism is to cross two oat lines, each monogenic for the genes in question, and look for segregation in a large F_2_ population. If there is no segregation, one can conclude either tight linkage or allelism. Currently, about 20 of the 90 known genes have been demonstrated to be either linked or allelic (in different groups). The biggest group of linked genes includes *Pc40*, *Pc44*, *Pc46*, *Pc50*, *Pc68*, *Pc95*, *PcX*, *Pg3*, and *Pg9*. Then there are the smaller groups of *Pc38*, *Pc62*, and *Pc63*, and *Pc35* and *Pc54* (Simons *et al.* 1978). However, testing allelism of all *Pc* genes would require numerous crosses (*i.e.*, a diallel crossing scheme) and phenotyping hundreds of thousands of F_2_ plants. Another significant difficulty is that some *Pc* genes are complex. More recent examples are *Pc58* and *Pc59* from *A. sterilis*. *Pc58* is actually a complex of three linked genes (Hoffman *et al.* 2006) and the *Pc59* differential line carries three unlinked *Pc* genes (Bush and Wise 1996). Future research could definitively resolve whether *Pc45* and *PcKM* are allelic or tightly linked. For instance, they could be mapped to different genetic intervals in large mapping populations (high resolution/fine mapping). Or they could be demonstrated to be the same gene by cloning one gene and sequencing the gene in carriers of the other gene and knockout mutants.

The *Pc45* gene was first used as a crown rust differential for race identification in Canada in 1974 (Harder 1975). This single-gene line continued to be useful as crown rust differential with the North American system of nomenclature for *P. coronata races* because of its differential response to various crown rust populations in North America (Chong *et al.* 2000, Carson 2008). Virulence to *Pc45* was either absent or at a very low frequency in Canada and in the main winter and spring oat regions in the US (Carson 2008). This gene has never been known to be deployed in commonly grown cultivars. Interestingly, rust surveys conducted in oat growing regions of the US from 2006 to 2009 showed high incidence of races virulent to *Pc45* reaching a frequency of up to 37% (Carson 2011). Similar findings were observed in the prairie regions of Canada from 2008 to 2017 (Chong *et al.* 2011, Menzies unpublished data) in which the incidence of virulence to *Pc45* has reached frequencies of more than 60%.

Carson (2011) reported that virulence to *Pc45* was highly correlated (*P* < 0.001) with virulence to the crown rust resistance in the oat line IA B605-X among 571 single-pustule isolates of oat crown rust. IA B605-X is a genetically uncharacterized isoline developed by Iowa State University and is in the pedigree of Kame and Morton. IA B605-X is believed to have contributed the *PcKM* gene to those cultivars from an unknown *A. sterilis* accession. The correlation in virulence to *Pc45* and *PcKM*, similar low infection types of *Pc45* and *PcKM*, and identical linkage map locations is strong evidence supporting that *Pc45* and *PcKM* are the same gene.

The SNP avgbs2_172881.1 was the closest GBS-derived marker to *Pc45/PcKM* and was closer to the gene than the previously identified marker I05-0874-KOM16c1 in the mapping populations despite the lower predictive power in the haplotype panel. Avgbs2_172881.1 was as predictive as GMI_ES02_c16987_268, but was a dominant marker that could not differentiate between homozygous resistant and heterozygous individuals. The combinations of I05-0874-KOM16c1 with avgbs2_172881.1 or GMI_ES02_c16987_268 identify a unique haplotype consistent with the presence of *Pc45* and *PcKM*.

Oat breeders need to be aware that *Pc45* and *PcKM* are most likely the same gene and thus cannot be pyramided. If they are tightly linked genes, then a rare recombinant would be required to put them in coupling. However given the correlated virulence to these genes in the pathogen population, pyramiding them would have little benefit for controlling crown rust. The findings of this study emphasize the need to map the known crown rust resistance genes to fully characterize the allelic relationships between *Pc* genes.
